# Myocardial T1 and extracellular volume fraction mapping at 3 tesla

**DOI:** 10.1186/1532-429X-13-75

**Published:** 2011-11-28

**Authors:** Jason J Lee, Songtao Liu, Marcelo S Nacif, Martin Ugander, Jing Han, Nadine Kawel, Christopher T Sibley, Peter Kellman, Andrew E Arai, David A Bluemke

**Affiliations:** 1 Radiology and Imaging Sciences, National Institutes of Health Clinical Center, Bethesda, MD, USA; 2 Molecular Biomedical Imaging Laboratory, National Institute of Biomedical Imaging and Bioengineering, Bethesda, MD, USA; 3 Laboratory of Cardiac Energetics, National Heart, Lung and Blood Institute, Bethesda, MD, USA; 4 U.S. Food and Drug Administration, Rockville, MD, USA

## Abstract

**Background:**

To compare 11 heartbeat (HB) and 17 HB modified lock locker inversion recovery (MOLLI) pulse sequence at 3T and to establish preliminary reference values for myocardial T1 and the extracellular volume fraction (ECV).

**Methods:**

Both phantoms and normal volunteers were scanned at 3T using 11 HB and 17 HB MOLLI sequence with the following parameters: spatial resolution = 1.75 × 1.75 × 10 mm on a 256 × 180 matrix, TI initial = 110 ms, TI increment = 80 ms, flip angle = 35°, TR/TE = 1.9/1.0 ms. All volunteers were administered Gadolinium-DTPA (Magnevist, 0.15 mmol/kg), and multiple post-contrast MOLLI scans were performed at the same pre-contrast position from 3.5-23.5 minutes after a bolus contrast injection. Late gadolinium enhancement (LGE) images were also acquired 12-30 minutes after the gadolinium bolus.

**Results:**

T1 values of 11 HB and 17 HB MOLLI displayed good agreement in both phantom and volunteers. The average pre-contrast myocardial and blood T1 was 1315 ± 39 ms and 2020 ± 129 ms, respectively. ECV was stable between 8.5 to 23.5 minutes post contrast with an average of 26.7 ± 1.0%.

**Conclusion:**

The 11 HB MOLLI is a faster method for high-resolution myocardial T1 mapping at 3T. ECV fractions are stable over a wide time range after contrast administration.

## Background

Cardiovascular magnetic resonance (CMR) is a noninvasive imaging method for accurate assessment of myocardial anatomy and function. A unique feature of CMR is the ability to use the proton relaxation times, such as T1, T2, and T2*, to characterize myocardial or vascular tissue [1]. These values are largely dependent on the physical and chemical environments of water protons in the tissue. Myocardial fibrosis is one of the most common histological features of the failing heart [2]. Late gadolinium enhancement (LGE) has been the standard of reference for detecting focal myocardial fibrosis in clinical practice [3]. LGE relies on the differences in signal intensity between scarred and adjacent normal myocardium to generate image contrast. Because this method lacks a normal myocardium reference value, LGE is unlikely to detect the presence of diffuse myocardial fibrosis tissue. T1 mapping, is a promising quantitative method for detecting diffuse myocardial fibrosis.

The Modified Look-Locker Inversion-recovery (MOLLI) technique uses electrocardiogram (ECG)-gated image acquisition at end-diastole and merges images from multiple consecutive inversion-recovery experiments into one data set. A high-resolution T1 map of the myocardium can be acquired in one breath hold [4,5]. The traditional MOLLI protocol uses three inversion-recovery blocks to acquire 11 images over 17 heartbeats (HB). These longer breath holds may limit its clinical applications in patients with impaired respiratory conditions due to cardiac diseases. Several new protocols have been proposed to reduce the MOLLI acquisition time [6,7].

Post contrast myocardial T1 values are affected by a variety of factors. These include, but are not limited to, the type and amount of gadolinium contrast injected, post contrast scan times, renal function, hematocrit, B_o _field, body composition, as well as pre-contrast T1 time. T1 mapping must take into account corrections for these variables before further analyses can be made [8]. Normalizing myocardial T1 in relationship to blood T1 avoids much of these complexities. Extracellular volume fraction (ECV), fibrosis index [9], or volume of distribution [10,11] are similar methods that measure the myocardial extracellular volume fraction by normalizing myocardial R1 change with blood R1 change and by correction for hematocrit. Although previous studies show that myocardial ECV quantifications correlate with diffuse myocardial fibrosis [10,12], these values have not yet been reported for 3T CMR.

In this study, we evaluated the accuracy of the 11 HB and 17 HB MOLLI sequences at 3T through phantom and normal volunteer studies. Preliminary reference values of both pre-contrast and post-contrast were quantified at different time points. Additionally, preliminary reference ECV values were also estimated from the normal volunteers.

## Methods

All experiments were performed at a 3 Tesla scanner (Verio, Siemens Medical Systems, Erlangen, Germany) with a 32-channel cardiovascular array coil (Invivo, Orlando, FL).

### I. CMR parameters

The 17 HB MOLLI consists of three inversion blocks: 3 images are acquired after each of the first two inversion pulses and 5 images are acquired after the third inversion pulse. The 11 HB MOLLI consists of 2 inversion blocks: 3 images are acquired after the first inversion pulse and 5 images are acquired after the second inversion pulse. Both the 17 HB and 11 HB protocol share all other parameters: 3 heartbeat pause between inversion blocks, nonsegmented steady state free precession readout, field of view: 360 × 290 mm, matrix: 256 × 180, slice thickness 10 mm, TR/TE: 1.9/1.0 ms, minimum inversion time 110 ms, inversion time increment 80 ms, flip angle 35 degrees, GRAPPA parallel imaging factor 2.

### II. Phantom Studies

The phantoms containing agarose gel doped with cupric sulfate were scanned using both 17 HB and 11 HB MOLLI sequence with simulated heart rates from 40 to 110 beats per minute (BPM) in increments of 10 heartbeats. To determine the reference T1 time of each phantom, standard inversion-recovery spin echo sequence with the same FOV and matrix size were acquired at 20 different TIs with a TR of 10 sec, and a TE of 8.5 ms.

### III. Human Volunteer Studies

Eleven healthy human subjects (six male and five female, ages 36 ± 13 years) without cardiovascular disease were enrolled for this study. All participants have normal ECG and were briefed on the procedure they were to undertake and all volunteers gave institutional review board approved written informed consent. 11 HB and 17 HB MOLLI images were acquired at mid-ventricular short axis view. All subjects were administered Gadolinium-DTPA contrast (Magnevist, 0.15 mmol/kg) at an injection rate of 2 ml/s followed by a 20 ml saline flush. Multiple post-contrast MOLLI scans were performed at the same pre-contrast position from 3.5, 5, 8.5, 13.5, 18.5 and 23.5 minutes after contrast injection. Standard phase sensitive inversion recovery late gadolinium enhancement imaging was acquired at 12-30 minutes after contrast injection [13]. Blood samples were taken 1 to 4 hours prior to the CMR scan to determine the hematocrit and creatinine.

### IV. Image Analysis

For both phantom and human studies, T1 maps were calculated using MRmap [14] and stored in Digital Imaging and Communications in Medicine (DICOM) Format. To extract myocardial T1 values for the human subjects, endocardial and epicardial contours were manually traced using QMass MR 7.2 (Leiden, Netherlands). The blood pool regions were carefully excluded when the regions of interest were traced. Phantom MOLLI T1 values of different heart rates were fitted to IR-SE T1 values by 2^nd ^order polynomial function and pre-contrast *in-vivo *MOLLI T1 values were corrected according to the phantom result. ECV values were calculated using [11]:

$$\begin{array}{l} \Delta {\text{R1}}_{\text{myo}}=\text{1}/{\text{T1}}_{\text{myo}-\text{post}}–\text{1}/{\text{T1}}_{\text{myo}-\text{pre}}\\ \Delta {\text{R1}}_{\text{blood}}=\text{1}/{\text{T1}}_{\text{blood}-\text{post}}–\text{1}/{\text{T1}}_{\text{blood}-\text{pre}}\\ \text{ECV}=\Delta {\text{R1}}_{\text{myo}}/\Delta {\text{R1}}_{\text{blood}}\times \left(\text{1}00-\text{HCT}\right) \end{array}$$

where ECV and HCT are give as percentage.

### V. Data Analysis

Statistical analyses were performed using SAS 9.1(Cary, North Carolina, USA) and Medcalc 11.6 (Medcalc Software, Belgium). Bland-Altman plots were used to describe the difference of T1 values between 17 HB and 11 HB protocols. To statistically compare the T1 values of two protocols, a general linear mixed model was used. The MOLLI protocol was included in the model as a fixed effect, while the phantom was included as a random effect. Similarly, all pre- and post-contrast 17 HB MOLLI T1, 11 HB MOLLI T1 as well as all ECV values quantified from volunteer data were also compared using a general linear mixed model. This model included time and the MOLLI protocols as fixed effects and the patients as random effect. The recovery rates of both the myocardial and blood pool post-contrast were compared using the Patrick Royston's p-trend test.

## Results

### I. Phantom

**Figure 1:**
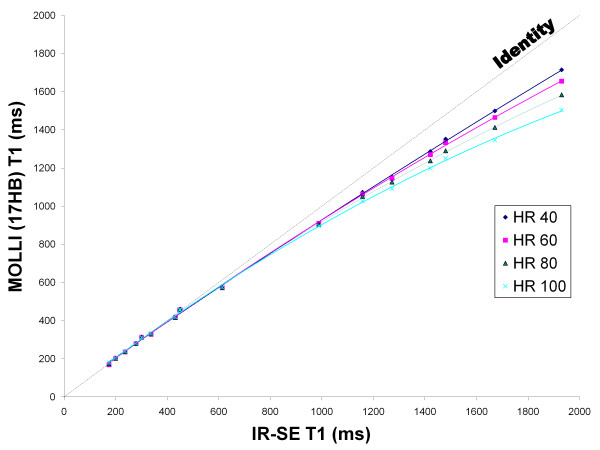
**Phantom Study**. 17 HB MOLLI sequences progressively underestimated T1 compared to IR-SE in phantoms as T1 and heart rate increased.

**Figure 2:**
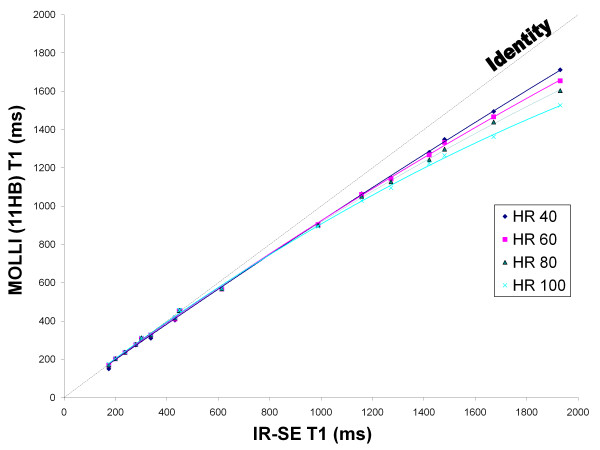
**Phantom Study**. 11 HB MOLLI sequences progressively underestimated T1 compared to IR-SE in phantoms as T1 and heart rate increased.

**Figure 3:**
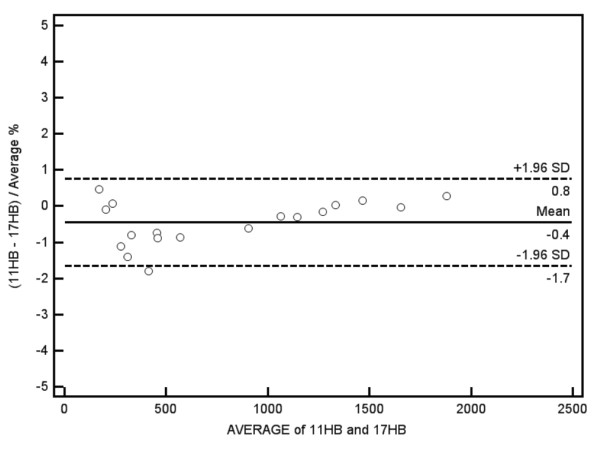
**Phantom Study**. Bland-Altman Plot comparing 17 HB MOLLI and 11 HB MOLLI T1 values at 60 bpm.

Both 11 HB and 17 HB MOLLI results were similar to IR-SE T1 values up to ~500 ms but both sequences underestimated T1 time for values > 500 ms. This underestimation was non linear and increased with higher heart rates. The 17 HB MOLLI acquires 11 images whereas the 11 HB MOLLI acquires 8 images. By comparing both sequences, the 11 HB MOLLI has a predicted noise penalty of 17% compared to the 17 HB MOLLI ($\sqrt{\left(11/8\right)}$). Comparing both sequences, the acquisition time was reduced by 35% when using the 11 HB MOLLI. Figure 1 and 2 respectively display the 17 HB and 11 HB MOLLI results compared to IR-SE. There was no significant difference between the 11 HB and 17 HB MOLLI (p = 0.82) by the general linear mixed model. A Bland-Altman plot comparing the two sequences at 60 bpm is presented in Figure 3 and displays a mean difference of 0.45 ± 1.96%. There is no significant bias and no relationship between the magnitude of T1 and error.

### II. Volunteers

**Figure 4:**
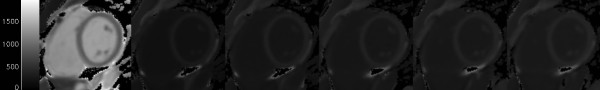
**Human Study**. Mid-ventricular short axis T1 maps. From left to right: pre-contrast, 5 minutes, 8.5 minutes, 13.5 minutes, 18.5 minutes and 23.5 minutes post-contrast. All images were displayed with same setting.

All volunteers had normal renal function (eGFR: 108 ± 17 ml/min/1.73 m^2^) and no focal myocardial scars were detected on the LGE images. The pre- and post-contrast MOLLI images were all interpretable for T1 mapping and contained little to no artifacts. Figure 4 shows a mid-ventricular short axis T1 map for pre- and post-contrast. There was no significant difference between 11 HB and 17 HB MOLLI in volunteers (p = 0.41) by the general linear mixed model. All values reported in this study were therefore based on measurements acquired using the 11 HB MOLLI only.

**Figure 5:**
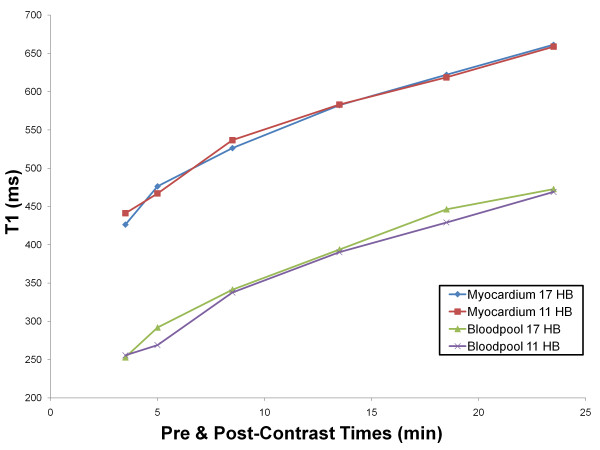
**Human Study**. Recovery of absolute myocardial and blood T1 from 3.5 minute to 23.5 minutes after 0.15 mmol/kg of Gd-DTPA.

T1 values are field strength dependent and increase with field strength [15]. The mean pre-contrast T1 values of myocardium and blood were 1315 ± 39 ms and 2020 ± 129 ms respectively. A post-contrast myocardium and blood T1 plot is presented in Figure 5. This plot demonstrates that gadolinium contrast is at dynamic equilibrium between myocardium and blood pool where their respective T1 values are recovering at a similar rate from 3.5 to 23.5 minutes (p = 0.86).

**Figure 6:**
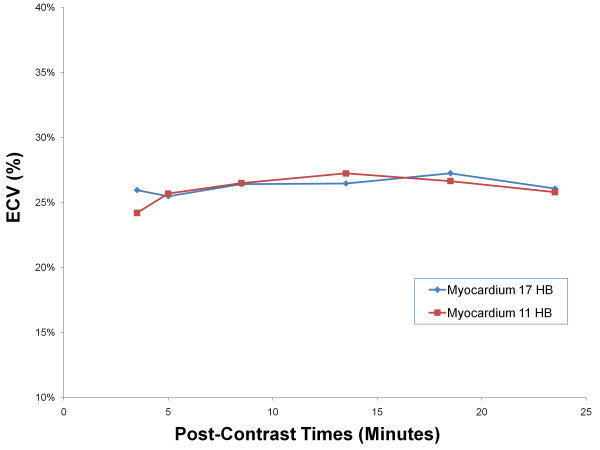
**Human Study**. Average ECV fractions of post-contrast times for both 17 HB MOLLI and 11 HB MOLLI.

**Figure 7:**
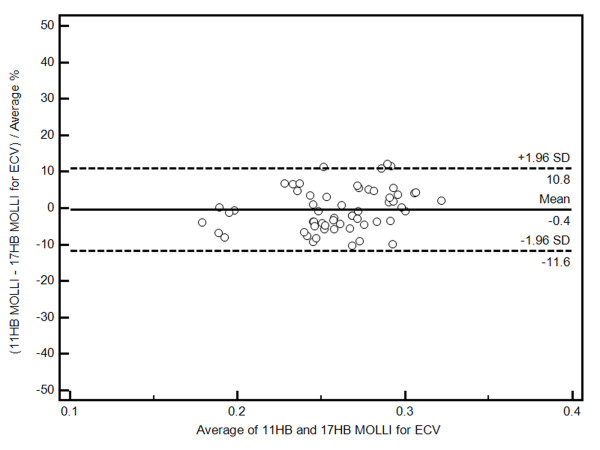
**Human Study**. Bland-Altman plot comparing all 17 HB MOLLI and 11 HB MOLLI ECV.

ECV was calculated at all post contrast time points by normalizing myocardial R1 change with blood R1 change and also correcting for hematocrit. Figure 6 displays the mean ECV values for each post-contrast time interval. There was no significant difference between 11 HB and 17 HB ECV in volunteers (p = 0.35). In addition, there is no significant difference of ECV after 8.5 minutes (p = 0.10). The least square mean of ECV between 8.5 minutes and 23.5 minutes was 26.7 ± 1.0%. A Bland-Altman plot comparing the two sequences for all ECV values is presented in Figure 7.

Table 1 display myocardial and blood pool T1 values as well as myocardial ECV values using both the 17 HB and 11 HB MOLLI sequences. The standard deviations (SD) for all T1 and ECV values are also presented.

**Table 1:** Myocardial and blood pool T1 as well as myocardial ECV values using both the 17 HB and 11 HB MOLLI sequences at different time points (Average ± standard deviation)

Structure	Sequence	Pre-Contrast	Post 3.5 min	Post 5 min	Post 8.5 min	Post 13.5 min	Post 18.5 min	Post 23.5 min
**Myocardium T1 (ms)**	17 HB MOLLI	1,324 ± 48	426 ± 44	476 ± 42	526 ± 31	583 ± 43	622 ± 40	661 ± 38
**Myocardium T1 (ms)**	11 HB MOLLI	1,314 ± 39	441 ± 50	467 ± 43	537 ± 52	583 ± 48	619 ± 48	659 ± 52
**Blood pool T1 (ms)**	17 HB MOLLI	2,037 ± 152	246 ± 33	282 ± 38	331 ± 37	384 ± 38	433 ± 42	462 ± 41
**Blood pool T1(ms)**	11 HB MOLLI	2,020 ± 128	255 ± 39	269 ± 22	337 ± 43	390 ± 44	429 ± 39	469 ± 42
**Myocardium ECV (%)**	17 HB MOLLI	N/A	25.5 ± 4.1	26.4 ± 3.8	26.5 ± 3.8	27.3 ± 4.2	26.1 ± 3.4	26.3 ± 3.8
**Myocardium ECV (%)**	11 HB MOLLI	N/A	24.2 ± 3.8	25.7 ± 3.8	26.5 ± 4.4	27.2 ± 4.6	26.7 ± 3.9	25.8 ± 4.1

## Discussion

The MOLLI sequence has been demonstrated to be an accurate and reproducible approach for myocardial T1 mapping [4,5]. The originally described MOLLI protocol requires a 17 heartbeat breath hold. A breath hold duration of 17 heart beats is difficult to achieve for older subjects and patients with impaired cardiovascular or pulmonary function. In this study, we compared a two inversion-recovery block 11 heartbeat MOLLI with the classic three inversion-recovery block 17 heartbeat MOLLI. With a 35% reduction in acquisition time, the 11 HB MOLLI achieved excellent agreement with the 17 HB MOLLI over a wide range of T1 times in our phantom study. There was no statistical difference between the two protocols for normal subjects for both pre-contrast and a series of post-contrast time points at 3T. The 11 HB MOLLI protocol is easy to implement on the scanner and requires no modification by post processing software. Both the 11 HB MOLLI and 17 HB MOLLI exhibited a systemic, nonlinear heart rate dependency of longer T1 values [5]. The observed deviation in measured versus actual T1 with lengthening T1 and shortened RR interval is as expected and does not display an inconsistent relationship. A heart rate correction of pre-contrast myocardial and blood T1 values is thereby recommended at 3 Tesla. Compared to shorter (and accurate) post-contrast T1's, the longer (and inaccurate) pre-contrast T1 times translated to R1 (1/T1) have less effect on the ECV.

For T1 mapping to be a routine clinical tool, the 17 HB MOLLI protocol must have a significantly reduced acquisition time. *Piechnik et al *[6] proposed a 9 heartbeat shortened MOLLI (shMOLLI). Their sequence is a 3 inversion-recovery block scheme that collects 7 images in 9 heartbeats. Because of the insufficient magnetization recovery of the last 2 inversion-recovery blocks, the last 2 images were only used to fit the short T1 times by using a conditional fitting algorithm. The shMOLLI results displayed good correlation with T1 times less than 800 ms, longer T1 times were underestimated about 4%.

Imaging at 3T has become the standard for neurological and musculoskeletal imaging. Cardiac imaging has been slow to adopt 3T imaging because of the inhomogeneities of both static magnetic field (B_0_) and the transmit radiofrequency field (B_1_) [16]. With the recent technological development of parallel radiofrequency transmission [17] as well as improved shimming algorithms [18], 3T scanners have been a more viable option for cardiac imaging because of the improvements in signal-to-noise ratio.

Compared to limited literature reports, the pre-contrast blood T1 values measured in our study (2020 ms) showed similar mean values with the results presented in a study conducted by *Stanisz et al *[19] (1932 ms). In contrast, the results for both these studies are significantly longer than the results of studies conducted by *Sharma et al *[20] (1670 ms) and *Noeske et al *[16] (1550 ms). This difference can be attributed to the limited range of TI values for these studies (100-800 ms and 500-1500 ms, respectively). The pre contrast blood T1 values from our data are about 26% to 38% longer than those of 1.5T results. For pre-contrast myocardial T1 times, our results (1315 ms) are shorter than those obtained by *Stanisz et al *[19] (1471 ms), but longer than the results acquired by *Piechnik et al *[6] (1169 ms), *Sharma et al *[20] (1200 ms) and *Noeske et al *[16] (1115 ms). Compared to previous myocardial T1 data at 1.5T, the pre-contrast myocardial T1 at 3T are 22% to 33% greater.

Pre-contrast T1s are longer at 3T and may not fully recover when the same sampling schemes are used as for 1.5T. A study conducted by *Schelbert et al *[7] introduced a hybrid MOLLI protocol adapted to the expected pre-contrast or post-contrast T1 values. At 1.5T, longer pre-contrast T1 times used a 2 inversion-recovery block scheme that collects 6 images. The shorter post-contrast T1 times used a 3 inversion-recovery block scheme that collects 7 images to cover for faster relaxation rates. Both the pre- and post-contrast scans were acquired in 11 HB. The hybrid MOLLI exhibits less bias and better agreement with the classic 17 HB MOLLI gold standard values at 1.5T. *Breton et al *[21] proposed a two heart beat saturation recovery T1 mapping technique. By acquiring a proton density image and a 500 ms saturation recovery image, T1 maps were generated by solving the Bloch equation. Although this method has very high acquisition speeds which effectively eliminate cardiac motion artifacts, it suffers from limited spatial resolution and limited T1 accuracy.

Extracellular volume fraction (ECV) [22,23], volume of distribution (Vd)[10], fibrosis index [24], and volume fraction of extravascular extracellular matrix (Ve) [7] all share the same parameters for measuring the myocardial extracellular matrix by adjusting the gadolinium contrast partition coefficient with the patient's hematocrit. A study conducted by *Kehr et al *[24] demonstrated that gadolinium distribution volume is closely correlated with histological collagen volume fraction (CVF) *in vitro*. Messroghli et al [12] recently reported there is a moderate correlation (r = 0.69) between CMR ECV and histological collagen volume fraction in a small animal model of left ventricular hypotrophy.

To accurately quantify ECV, steady state equilibrium of gadolinium chelates must be reached between the plasma and myocardial interstitium. Following an intravenous injection, gadolinium chelates such as Gd-DTPA are continuously cleared from the blood via renal clearance. After an initial period of equilibration, the contrast agent concentration in the blood will steadily decrease over time. However, if the contrast exchange rate between blood and tissue is faster than the renal clearance, then the ratio of contrast agent concentration between tissue and blood will, after the short initial equilibration period, achieve a dynamic equilibrium over a certain time [11], (Ugander *et al*, Extracellular volume imaging by MRI provides insight into overt and subclinical myocardial pathology, in press). *Flett et al *[10] developed the equilibrium contrast CMR method (EQ-CMR), by administering contrast via bolus injection followed by a slow continuous infusion. Their results showed that volume of distribution correlates with histological CVF in aortic stenosis and hypertrophic cardiomypathy. However, longer infusion methods are difficult to incorporate into clinical CMR workflow. A previous study completed by *Schelbert et al *[7] validated the bolus technique as an equally accurate method as the slow infusion technique for quantifying ECV. We have verified this dynamic equilibrium in normal volunteer at 3T by repeatedly measuring both myocardial and blood T1 at different time points. In certain pathological situations, such as the microvasular obstruction, the assumption of dynamic equilibrium is not valid.

Our study used the bolus injection technique. General linear mixed model demonstrated that there is no statistical difference of ECV values from 8.5 minutes. Between 8.5 to 23.5 minutes, the ECV differences between consecutive time points were less than 3%. This difference is significantly less than the coefficient of variation for our average ECV value. Compared to previous reports, our results suggest that the gadolinium concentration could reach dynamic equilibrium between plasma and myocardium as early as 8.5 minutes. Combining our results with those of *Schelbert et al *[7], ECV is stable in normal volunteers from 8.5 to 50 minutes after bolus contrast injection. Our findings permit considerable flexibility for incorporating ECV into routine CMR work flow. A limitation of both this study and Schelbert's study is that there is a lack of substantial data for subjects with cardiac disease. Diseased subjects may have areas of severe fibrosis/scarring or cardiac dysfunction. Either of these conditions could delay the time to reach steady-state equilibrium. Further testing in patients with severe fibrosis and reduced ejection fraction should be done to verify whether continuous infusions can be fully replaced by the single-bolus approach in clinical settings.

T1 values are affected by confounding variables such as field strength, gadolinium contrast type and dose, scanning time and the patient's renal function. Due to these factors, T1 times cannot be readily compared to T1 data from other centers. In contrast, ECV is an inherent physiological property that should not be affected by these variables. The ECV data of normal volunteers from our study at 3T (26.7%) slightly higher than the "fibrosis index" of normal volunteers reported by *Broberg et al *[9] (24.8%) and the Ve quantified by *Schelbert et al *[7] after the Ve value was converted into comparable ECV values (24.1%). The higher ECV seen in this study might be an effect of relatively stronger shortening of the longer pre-contrast blood T1 at 3T as compared to that at 1.5 T. In addition, Both Broberg and Schelbert's study used protocols significantly different from the protocol used in our study.

## Conclusions

In conclusion, we have validated MOLLI sequences at 3T in phantom studies. We present values for myocardial and blood T1 pre and post gadolinium contrast at 3T. At 3T, post-gadolinium ECV is stable between 8.5 and 23.5 minutes after gadolinium injection.

## Limitations

There are several limitations to this study. This studied had a small number of subjects with varying ages and genders. Only a single contrast agent was used for this study. There is no direct comparison to EQCMR or ShMOLLI. There is no repeatability analysis with intra and inter-observer variability. Only the mid-cavity ventricular slice was taken and the mean T1 for the entire myocardium was used. There may be some variations in T1 and ECV in the basal, apical, septal and lateral walls.

## Competing interests

The authors declare that they have no competing interests.

## Authors' contributions

All authors read, critically edited the initial manuscript, added intellectual content, and approved the final version. DAB and SL designed, coordinated and conducted the study, MU created the phantoms. SL and MSN acquired all data, JJL and NK analyzed all data, JH conducted the statistical analyses. PK assisted with pulse sequence optimization. AEA assisted with the study design, data analysis, and added critical manuscript content.
